# Association between MCP-1 -2518A/G Polymorphism and Cancer Risk: Evidence from 19 Case-Control Studies

**DOI:** 10.1371/journal.pone.0082855

**Published:** 2013-12-18

**Authors:** Liang-Shan Da, Ying Zhang, Shuai Zhang, Yi-Chun Qian, Qin Zhang, Feng Jiang, Lin Xu

**Affiliations:** 1 The First Clinical College of Nanjing Medical University, Nanjing, Jiangsu, China; 2 Department of Thoracic Surgery, Nanjing Medical University Affiliated Cancer Hospital Cancer Institute of Jiangsu Province, Nanjing, Jiangsu, China; 3 Wannan Medical College, Wuhu, Anhui, China; 4 Department of Ultrasonic Medicine, Wannan Medical College Affiliated Yijishan Hospital, Wuhu, Anhui, China; MOE Key Laboratory of Environment and Health, School of Public Health, Tongji Medical College, Huazhong University of Science and Technology, China

## Abstract

**Background:**

Single nucleotide polymorphisms (SNPs) may affect the development of diseases. The -2518A/G polymorphism in the regulatory region of the monocyte chemo-attractant protein-1 (MCP-1) gene has been reported to be associated with cancer risk. However, the results of previous studies were inconsistent. Therefore, we performed a meta-analysis to obtain a more precise estimation of the relationship between the -2518A/G polymorphism and cancer risk.

**Methodology/Principal Findings:**

We performed a meta-analysis, including 4,162 cases and 5,173 controls, to evaluate the strength of the association between the −2518A/G polymorphism and cancer risk. Odds ratio (OR) and 95% confidence intervals (95% CIs) were used to assess the strength of association. Overall, the results indicated that the −2518A/G polymorphism was not statistically associated with cancer risk. However, sub-group analysis revealed that individuals with GG genotypes showed an increased risk of cancer in digestive system compared with carriers of the A allele (GG vs. AA: OR = 1.43, 95%CI = 1.05–1.96, P_heterogeneity_ = 0.08; GG vs. AG/AA: OR = 1.29, 95%CI = 1.02–1.64, P_heterogeneity_ = 0.14). In addition, the increased risk of GG genotype was also observed in Caucasians (GG vs. AG/AA: OR = 1.81, 95%CI = 1.10–2.96, P_heterogeneity_ = 0.02).

**Conclusion:**

This meta-analysis suggests that the MCP-1 −2518A/G polymorphism may have some relation to digestive system cancer susceptibility or cancer development in Caucasian. Large-scale and well-designed case-control studies are needed to validate the findings.

## Introduction

Cancer is a major public health problem and one of the principal causes of death worldwide [1]. It is predicted that the number of newly diagnosed cancers in the world will increase to more than 15 million and 12 million people will die of cancer in 2020[2]. It has been widely accepted that carcinogenesis is a consequence of complex inherited and environmental factors. However, the exact mechanism of carcinogenesis remains largely unknown. Epidemiological study points a connection between chronic inflammation and various cancers [3], and it is estimated that 15–20% of all deaths from cancer are associated with infections and inflammatory responses [4]

Monocyte chemo-attractant protein 1 (MCP-1), also known as CCL-2 (CC chemokine ligand 2), is a member of the CC chemokine family which plays an important role in inflammation, and is encoded by the CCL-2 gene which locates on 17q11.2-q12 [5]–[7]. MCP-1is involved in a series of diseases including rheumatoid arthritis, chronic obstructive pulmonary disease, cardiovascular disease, and cancer [8]. Being a chemokine, MCP-1 is largely produced by cancer cells and is responsible for the recruitment of macrophages to many kinds of tumors, including cancers of ovary, breast, bladder, lung, and cervix [9]-[13], and high concentrations of tumor-associated macrophages (TAMs) are linked to better tumor growth and progression as well as poor prognosis [14]. Therefore, MCP-1 may play a critical role in tumor initiation, promotion, and progression [15].

Several MCP-1 polymorphisms have been reported to be associated with disease susceptibility or severity [16], and the −2518A/G (rs1024611) polymorphism which can increase the expression of MCP-1 was most widely studied [6]. Recently, an increasing number of studies have examined the association between this −2518A/G polymorphism and cancer risk [15]–[32]. However, individual study may have insufficient power to obtain a comprehensive and reliable conclusion. We, therefore, performed a meta-analysis by pooling all eligible studies to clarify this inconsistency and to achieve a more precise estimation of the relationship between the MCP-1 −2518A/G polymorphism and cancer risk.

## Methods

### Identification and eligibility of relevant studies

A systematic search of PubMed and China National Knowledge Infrastructure (CNKI) database (last search updated in June 2013) was carried out to identify case-control studies that investigated the association between the −2518A/G polymorphism and cancer risk. The search strategy was based on combinations of “MCP-1”, “CCL-2”; “cancer”, “carcinoma”, “tumor”; “polymorphism”, “variant”, “SNP”. In order to minimize potential publication bias, citations in original studies were also screened by manual search to identify additional relevant publications. The selection criteria of the retrieved articles in our meta-analysis were as follows: (1) a case–control design; (2) investigating the −2518A/G polymorphism and cancer risk; (3) sufficient data available to calculate an odds ratio (OR) with 95% confidence interval (CI). The major reasons for exclusion of studies were (1) investigations in subjects with cancer-prone disposition; (2) overlapping data; (3) not published in English and Chinese.

### Data extraction

The following information was collected independently by two of the authors (Da and Zhang) for each eligible study: name of first author, published year, country of origin, ethnicity, source of control, cancer type, genotyping method, total number of genotyped cases and controls, genotype frequencies in cases and control, and Hardy–Weinberg equilibrium (HWE) of controls. Ethnicity was categorized as Asian, Caucasian and mixed population. Cancer types were classified as bladder cancer, prostate cancer, digestive system cancer (oral cancer, gastric cancer, colorectal and hepatocellular cancer), and other cancers. All studies were defined as hospital-based (HB) or population-based (PB) according to the source of control. The final results of data extraction were compared carefully, and any disagreements were discussed until reaching conformity on all items among all authors.

### Statistical analysis

For each study, deviation from HWE among controls was evaluated by Pearson's χ^2^-test and a *P*<0.05 was considered as significant disequilibrium. The strength of the associations between the −2518A/G polymorphism and cancer susceptibility was measured by OR with its 95%CI. The pooled ORs and the 95% CIs in each comparison were calculated using the following models: homozygote model (GG vs. AA), heterozygote model (AG vs. AA), dominant model (GG/AG vs. AA) and recessive model (GG vs. AG/AA), respectively. Between-study heterogeneity was assessed by the chi-square based Q test and the heterogeneity was found to be significant when *P*<0.10[33]. The summary ORs were calculated by the fixed-effects model (Mantel-Haenszel method) when the *P* value was >0.10. Otherwise, the random-effects model (DerSimoniane-Laird method) was utilized [34]. The Z test was applied to determine the significance of the pooled ORs. And a P<0.05 was considered significant. Sub-group analyses and meta-regression were carried out to explore the source of heterogeneity among variables, including ethnicity, cancer types, source of control and sample size (studies with more than 500 participants were defined as “large”, and studies with less 500 participants were defined as “small”), respectively. Sensitivity analyses were performed by sequentially removing individual study to evaluate the robustness of the overall estimate. Finally, publication bias was examined by Begg's funnel plot and the Egger's linear regression test, and a *P*<0.05 was considered to be representative of statistically significant publication bias [35]. All p-values were two sided, and any statistical tests for this meta-analysis were done with STATA statistical software (version 12.0; StataCorp, College Station, Texas USA).

## Results

### Characteristics of eligible studies

**Figure 1:**
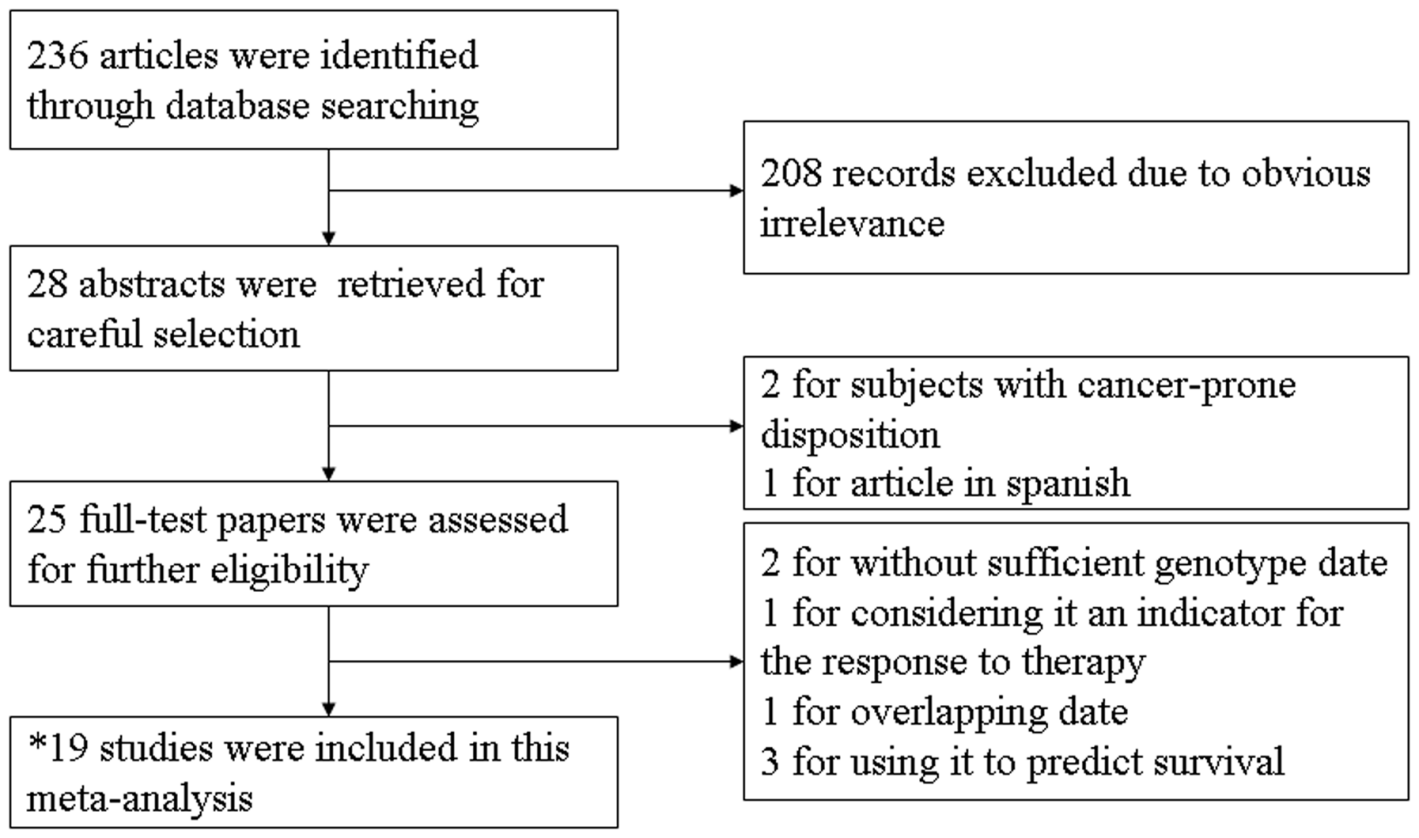
Flow diagram of the study selection process. *a total of 18 articles were identified and two types of cancers were reported in one article, we extracted data separately for each cancer, thus 19 studies were eligible.

After careful retrieve and selection, 18 eligible articles were identified according to inclusion and exclusion criteria. The study selection procedures were shown in Figure 1. Two types of cancers were reported in Qin's study, and we extracted data separately for each cancer. Therefore, a total of 19 case-control studies with 4,162 cases and 5,173 controls were included in this meta-analysis.

Out of the 19 applicable studies, 17 were published in English and 2 were written in Chinese, 10 of them were studies of Asians, 8 studies of Caucasian and one study of mixed population. According to the source of control, 10 studies were hospital-based and 9 were population-based. The genotype distributions in the controls were in agreement with HWE except for two studies (Gu [23], p<0.01; Attar [28], p = 0.04). The genotyping methods in studies were nearly all polymerase chain reaction-restriction fragment length polymorphism. The detailed characteristics of each case-control study were listed in Table 1.

**Table 1:** Characteristics of the eligible studies in the meta-analysis.

Study	Year	Country	Ethnicity	Cancer type	Control source	No. of case/control	Case			Control			HWE
							AA	AG	GG	AA	AG	GG	
Liu	2013	China	Asian	Renal	HB	416/458	59	197	160	93	234	131	Yes
Arshad	2013	India	Asian	Bladder	PB	120/190	32	64	24	60	87	8	Yes
Wu	2013	Taiwan	Asian	Cervical	HB	86/253	16	52	18	33	132	88	Yes
Kucukgergin	2012	Turkey	Caucasian	Bladder	HB	142/197	67	54	21	96	83	18	Yes
Singh	2012	India	Asian	Bladder	HB	200/200	83	101	16	81	97	22	Yes
Kuckergergin	2012	Turkey	Caucasian	Prostate	HB	156/152	78	67	11	64	71	17	Yes
Bektas-Kayhan	2012	Turkey	Caucasian	Oral	HB	129/140	67	56	6	94	45	1	Yes
Chen	2011	Taiwan	Asian	Oral	HB	216/344	49	112	55	80	172	92	Yes
Gu	2011	China	Asian	Gastric	HB	608/608	94	270	244	138	268	202	No
Kruszyna	2011	Poland	Caucasian	Breast	PB	160/323	89	54	17	154	145	24	Yes
Yeh	2010	Taiwan	Asian	Hepatocellular	HB	102/344	23	48	31	80	172	92	Yes
Yang	2010	China	Asian	Lung	PB	112/82	34	48	30	10	34	38	Yes
Narter	2010	Turkey	Caucasian	Bladder	PB	72/76	48	16	8	40	33	3	Yes
Attar	2010	Turkey	Caucasian	Endometrial	HB	50/211	26	17	7	124	82	5	No
Qin	2009	China	Asian	Hepatocellular	PB	397/471	133	182	82	185	225	61	Yes
Qin	2009	China	Asian	Nasopharyngeal	PB	575/471	185	299	91	185	225	61	Yes
Vazquez-Lavista	2009	Mexico	Mixed	Bladder	PB	47/126	9	35	3	18	71	37	Yes
Sáenz-López	2008	Spain	Caucasian	Prostate	PB	298/311	174	100	24	178	123	10	Yes
Landi	2006	Spain	Caucasian	Colorectal	HB	276/251	161	97	18	138	97	16	Yes

PB: population-based; HB: hospital-based; HWE: Hardy–Weinberg equilibrium

### Meta-analysis results

Overall, there was no statistically significant association between cancer risk and the −2518A/G polymorphisms in all genetic models (Table 2). However, strong evidence of heterogeneity was found in each comparison. Thus, sub-group analyses were performed to determine the influence of confounding factors.

**Table 2:** Meta-analysis of the association between the MCP-1 −2518A/G polymorphism and cancer risk in all genetic models.

		GG vs. AA		AG vs. AA		GG/AG vs. AA		GG vs. AG/AA	
	N	OR	P_h_	OR	P_h_	OR	P_h_	OR	P_h_
Total	20	1.28(0.95,1.73)	<0.001	1.00(0.86,1.16)	0.004	1.05(0.89,1.23)	<0.001	1.25(0.97,1.60)	<0.001
Cancer type									
Digestive system cancer	6	1.43(1.05,1.96)*	0.081	1.17(0.95,1.43)	0.149	1.24(0.99,1.56)	0.050	1.29(1.02,1.64)*	0.141
Bladder cancer	5	1.26(0.47,3.42)	<0.001	0.92(0.66,1.30)	0.124	0.99(0.70,1.39)	0.089	1.25(0.46,3.39)	<0.001
Prostate cancer	2	1.15(0.26,5.16)	0.008	0.81(0.62,1.07)	0.808	0.87(0.67,1.13)	0.337	1.27(0.30,5.39)	0.008
Others	6	1.13(0.59,2.16)	<0.001	0.90(0.63,1.29)	0.005	0.93(0.63,1.38)	<0.001	1.17(0.71,1.92)	<0.001
Ethnicity									
Asian	10	1.22(0.84,1.76)	<0.001	1.15(0.98,1.35)	0.142	1.16(0.94,1.43)	0.003	1.16(0.88,1.52)	<0.001
Caucasian	8	1.67(0.99,2.80)	0.012	0.85(0.67,1.07)	0.048	0.94(0.75,1.18)	0.040	1.81(1.10,2.96) *	0.015
Source of control									
PB	9	1.50(0.86,2.64)	<0.001	0.88(0.67,1.15)	0.003	0.97(0.74,1.27)	0.001	1.57(0.94,2.61)	<0.001
HB	10	1.12(0.80,1.58)	0.001	1.09(0.92,1.29)	0.152	1.11(0.90,1.36)	0.008	1.07(0.83,1.39)	0.005
Sample size									
Large^a^	7	1.59(1.29,1.96) *	0.173	1.13(0.96,1.34)	0.108	1.22(1.03,1.45) *	0.054	1.38(1.15,1.66) *	0.124
Small^b^	12	1.12(0.63,1.99)	<0.001	0.88(0.70,1.11)	0.026	0.91(0.71,1.17)	0.002	1.19(0.72,1.97)	<0.001

N: number of studies; OR: odds ratio; P_h_: p value for heterogeneity; *OR with statistical significance; PB: population-based; HB: hospital-based; a: studies with more than 500 participants; b: studies with less than 500 participants.

**Figure 2:**
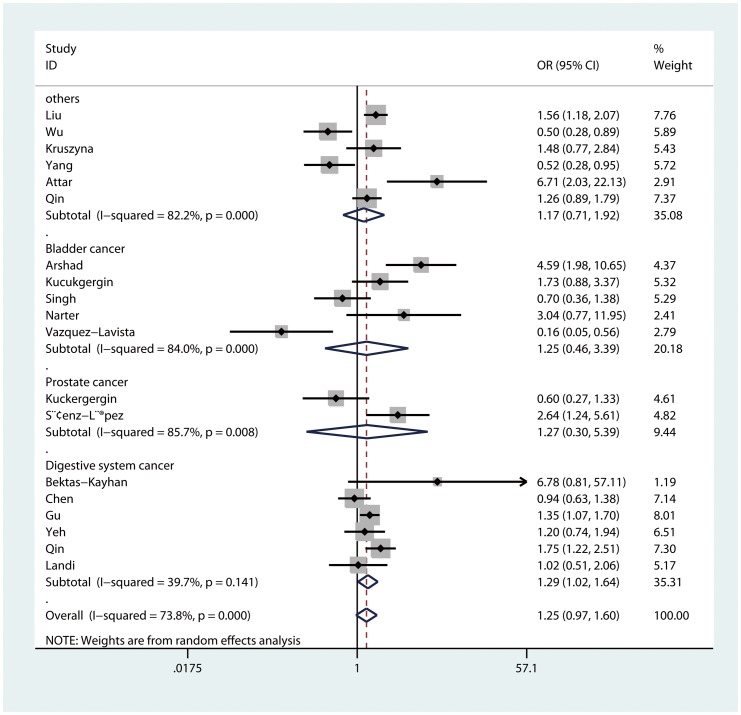
Forest plot of recessive model for overall comparison by cancer type (GG vs. AG/AA).

As for cancer type, a statistically increased cancer risk was found in the comparison of homozygote (GG vs. AA: OR = 1.43, 95%CI = 1.05–1.96, P_heterogeneity_ = 0.08) and recessive model (GG vs. AG/AA: OR = 1.29, 95%CI = 1.02–1.64, P_heterogeneity_ = 0.14, Figure 2) for digestive system cancer. However, no significant associations were discovered in bladder cancer, prostate cancer or other cancers.

**Figure 3:**
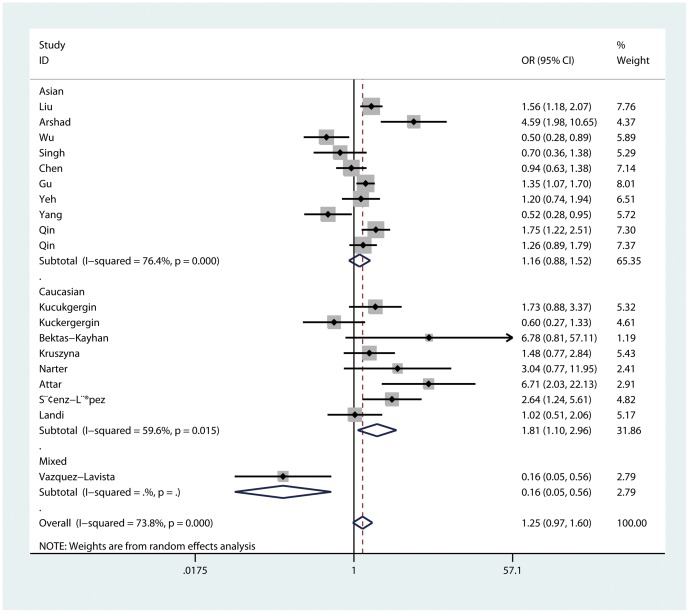
Forest plot of recessive model for overall comparison by ethnicity (GG vs. AG/AA).

When stratified by ethnicity, an increased cancer risk was found in the recessive model comparison for Caucasians (GG vs. AG/AA: OR = 1.81, 95%CI = 1.10–2.96, P_heterogeneity_ = 0.02, Figure 3), In Asians, however, no significant association but only a trend of increased cancer risk was found in each genetic model.

Further, in the stratified analyses by sample size and source of control, we observed a significantly increased risk in “large” studies in three genetic models: homozygote model (GG vs. AA: OR = 1.59, 95%CI = 0.74–2.05, P_heterogeneity_ = 0.17), recessive model (GG vs. AG/AA: OR = 1.38, 95%CI = 1.15–1.66, P_heterogeneity_ = 0.12) and dominant model (GG/AG vs. AA: OR = 1.22, 95%CI = 1.03–1.45, P_heterogeneity_ = 0.05). However, the cancer cases and controls did not significantly differ in the subgroup analyses according to the source of control.

### Evaluation of heterogeneity

Between-study heterogeneity was obvious in each model (Table 2). Meta-regression was further conducted to explore the sources of heterogeneity. The results indicated that cancer type (*P* = 0.02), but not ethnicity, source of control and sample size (*P*>0.05) contributed to source of heterogeneity.

### Sensitivity analysis and publication bias

**Figure 4:**
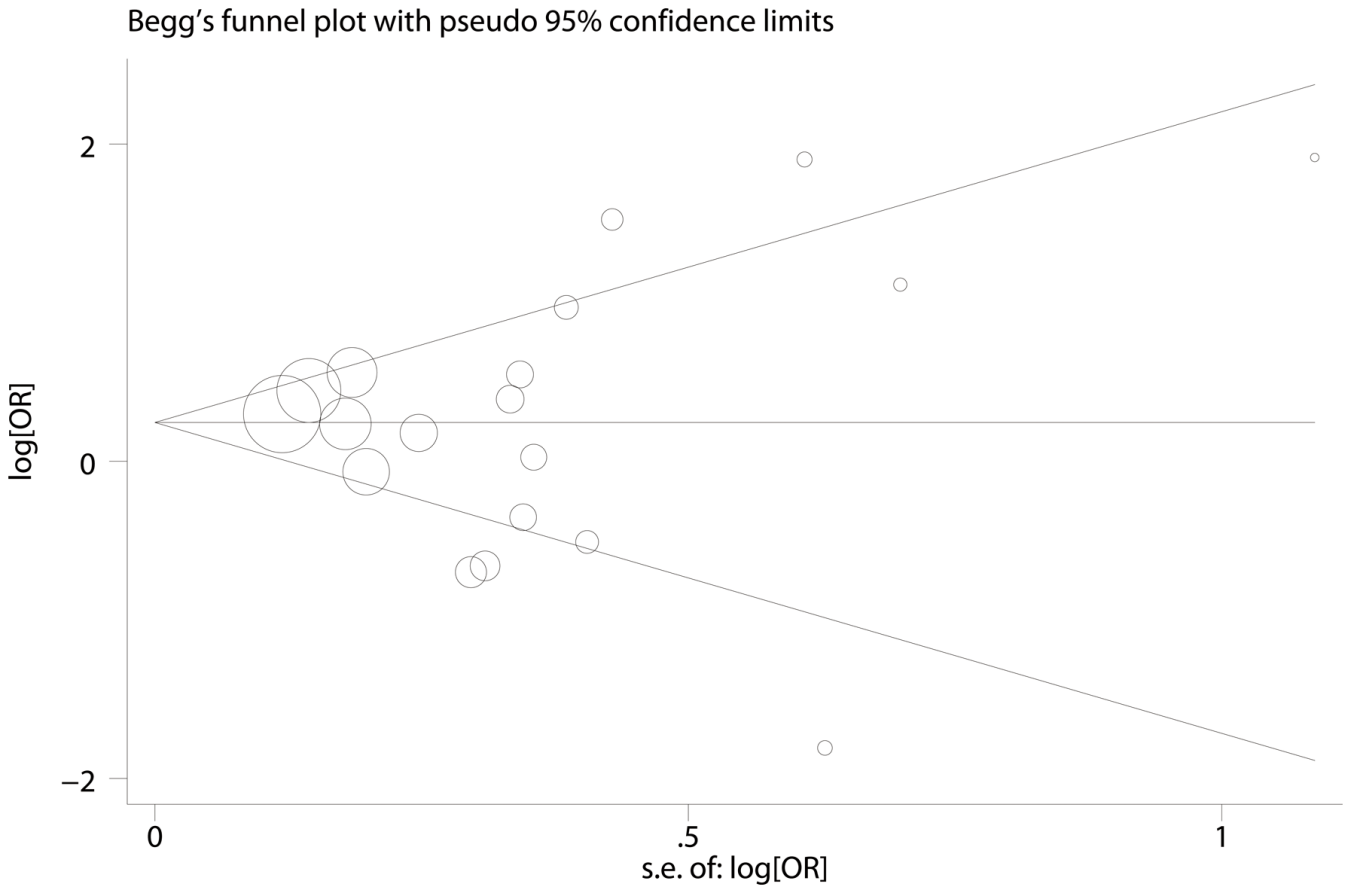
Begg's funnel plot of MCP-1 −2518A/G polymorphism and cancer risk (GG vs. AG/ AA).

A one-way sensitivity analysis was performed to assess the stability of the results of the meta-analysis. Statistically similar results were obtained after sequentially excluding individual studies, which confirmed the robustness of the meta-analysis (data not shown). For publication bias, as shown in Figure 4, the shape of the funnel plot did not reveal any evidence of obvious asymmetry(GG vs. AG/AA: *P* = 0.67), and the results of Egger's test also indicated no risk of publication bias (GG vs. AG/AA: *P* = 0.96)

## Discussion

The impacts of MCP-1 activation on tumor cells have been demonstrated in a variety of malignancies [8]. It has been shown that the −2518A/G SNP in the regulatory region of MCP-1 gene could affect transcription and increase the expression of MCP-1[6]. MCP-1 expression was associated with tumorigenesis and metastasis of several solid tumors [23]. The overexpression of MCP-1 has been reported in a wide range of tumors such as glioma, ovarian, esophageal, breast, lung, and prostate cancer [36]–[38]. In the light of these findings, it is reasonable that the −2518A/G polymorphism may contribute to cancer susceptibility. However, previous case-control studies have yielded inconsistent conclusions. In order to obtain a more precise estimation of this relationship, we performed this meta-analysis including 19 case-control studies with 4,162 cases and 5,173 controls, and the result demonstrated that the MCP-1 −2518A/G polymorphism was not associated with cancer susceptibility in overall analysis.

Sub-group analysis was conducted to detect the effects of confounding factors. When stratified by ethnicity, there was a significantly increased cancer risk among Caucasians but not in Asians. The differences may be explained by genetic diversities, different risk factors in life styles, and the exposure to different environmental factors. However, it was noteworthy that an increased cancer risk was found in the recessive model for Caucasians, and only two “large” studies were included in this subgroup. It was reported that small size may decrease statistical power and even may produce a fluctuated risk estimate. Therefore, this relationship needs to be further confirmed in larger size, well-designed prospective studies.

In the subgroup analysis by cancer type, no significant association was found except for homozygote model and recessive model comparison of digestive system cancer. This could be explained by the following two reasons: one may be that this polymorphism may play a different role in different cancer sites. The other possible reason is that most studies in this subgroup were “large” studies which have sufficient statistical power to investigate a slight effect compared with “small” studies. In consistent with this explanation, there was a significantly increased cancer risk in “large” studies in three genetic models, but no significant association was observed in “small” studies in any comparison.

Finally, attention should be paid to the relatively huge heterogeneity in this meta-analysis. Meta-regression indicated that cancer type (*P* = 0.02), but not ethnicity, source of control or sample size (*P*>0.05) contributed to the source of heterogeneity. In fact, numerous other factors including age, sex ratio, family history and lifestyle may also explain the heterogeneity. Unfortunately, we can not conduct a meta-regression utilizing these variables because detail information was not available.

Some limitations of this meta-analysis should be addressed. Firstly, only English and Chinese

papers were included in this meta-analysis,. Therefore, selection bias may have existed, although not any publication bias was showed in the funnel plot and Egger's tests. Secondly, this meta-analysis was based on unadjusted estimates, because adjusted estimates were not shown in all published studies. Thirdly, no genome-wide association studies (GWAS) date was included in this meta-analysis. As we know, as compared to the candidate-gene approach, GWAS have revolutionized the field of genetic susceptibility and provided a powerful approach to identify the common genetic variants. Therefore, this powerful and comprehensive approach have contributed to unprecedented advances in our understanding of the role of common genetic variation in various cancers[39]–[42].However, due to the strict criteria, some low-risk alleles might be overlooked in spite of their potential importance in disease risk.

In conclusion, this meta-analysis suggests that the MCP-1 −2518A/G polymorphism may have some relation to digestive system cancer susceptibility or cancer development in Caucasian. To further confirm the results, large scale case-control studies with different ethnic groups and multiple cancer types are needed.

## Supporting Information

Checklist S1 PRISMA checklist.
(DOC)
